# Sleep Health Inequities: Sociodemographic, Psychosocial, and Structural Determinants of Short Sleep in U.S. Adults

**DOI:** 10.3390/clockssleep7040059

**Published:** 2025-10-16

**Authors:** Lourdes M. DelRosso, Mamatha Vodapally

**Affiliations:** 1 Department of Medicine, Division of Pulmonary and Sleep, University of California, Fresno, CA 93701, USA; 2 Family Medicine Residency Program, Adventist Health Hanford Family Medicine, Hanford, CA 93230, USA; drmamathavodapally@gmail.com

**Keywords:** short sleep, BRFSS, social determinants of health, sleep disparities, psychosocial factors, health, public health

## Abstract

Short sleep duration (≤6 h) is a public health concern linked to cardiometabolic disease and premature mortality. However, persistent disparities across sociodemographic, psychosocial, and structural domains remain underexplored in recent nationally representative samples. We analyzed 2022 Behavioral Risk Factor Surveillance System (BRFSS) data, including 228,463 adults (weighted N ≈ 122 million). Sleep duration was dichotomized as short (≤6 h) versus adequate (≥7 h). Complex samples logistic regression estimated associations between sociodemographic, psychosocial, behavioral, and structural determinants and short sleep, accounting for survey design. The weighted prevalence of short sleep was 33.2%. Non-Hispanic Black (AOR = 1.56, 95% CI: 1.46–1.65) and American Indian/Alaska Native adults (AOR = 1.46, 95% CI: 1.29–1.65) were disproportionately affected compared with non-Hispanic White adults. Psychosocial factors contributed strongly: life dissatisfaction, limited emotional support, and low social connectedness increased odds, whereas high connectedness was protective. Food insecurity and smoking were significant structural and behavioral risks, while binge drinking and urbanicity were not. One-third of U.S. adults report short sleep, with marked disparities across demographic, socioeconomic status, psychosocial stressors, and structural barriers. Findings highlight the multifactorial nature of sleep health inequities and the need for multilevel interventions addressing both individual behaviors and upstream determinants.

## 1. Introduction

Sleep is a critical determinant of health and well-being and insufficient sleep is a major public health concern in the United States [1]. The American Academy of Sleep Medicine (AASM) recommends seven hours of sleep for adults, with sleep insufficiency, typically defined as fewer than 7 h per night [2]. Sleep Insufficiency or short sleep has been linked to numerous adverse outcomes, including obesity, cardiovascular disease, diabetes, depression, and premature mortality [3,4,5,6]. Short sleep duration also contributes to impaired productivity, reduced quality of life, and significant economic costs at both individual and societal levels [7,8].

Despite growing awareness, short sleep remains prevalent in the United States, with notable disparities across sociodemographic and psychosocial groups [9,10]. Prior studies have shown that racial and ethnic minority groups, individuals with lower income or education, and those living in disadvantaged environments are disproportionately affected by short sleep [11,12]. However, the strength and consistency of these associations vary across studies, and updated nationally representative estimates are needed. In addition, psychosocial stressors such as low life satisfaction, limited emotional support, social isolation, and financial insecurity may exacerbate sleep difficulties, though these factors have been less extensively studied at the population level [13,14,15].

The Behavioral Risk Factor Surveillance System (BRFSS) is a national health-related survey conducted by the U.S. Centers for Disease Control and Prevention (CDC), providing a unique opportunity to examine short sleep across diverse U.S. populations using a large, nationally representative dataset [16]. Using the BRFSS 2022 dataset, this study aims to estimate the prevalence of short sleep by sociodemographic and psychosocial characteristics, and evaluate its associations with multiple social determinants, including survey-specific variables such as income, education, marital status, smoking, alcohol use, race/ethnicity, and urban–rural status. Although the variable names follow CDC conventions, the term “race/ethnicity” is used as a social construct reflecting sociocultural and structural contexts rather than biological differences. We hypothesized that short sleep would be more common among racial and ethnic minorities, individuals of lower socioeconomic position, and those engaging in adverse health behaviors such as smoking and binge drinking.

## 2. Results

### 2.1. Sample Characteristics

The analytic sample included 228,463 adults from the 2022 BRFSS with valid data on sleep duration, representing a weighted U.S. population of approximately 122 million adults. Overall, the weighted prevalence of short sleep (<6 h) was 33.2%, while 66.8% reported adequate sleep (≥7 h). Respondents were predominantly non-Hispanic White (69.0%), female (52.3%), and college graduates (44.8%). The mean age was 54.1 years.

### 2.2. Bivariate Distributions

Prevalence of short sleep varied significantly across sociodemographic groups. Rates of short sleep were highest among adults with less than a high school education (42.5%) compared with college graduates (28.3%). Racial/ethnic disparities were pronounced: 46.0% of non-Hispanic Black adults and 44.8% of American Indian/Alaska Native adults reported short sleep, compared with 31.7% of non-Hispanic White adults. Adults who were divorced, separated, or widowed had a higher prevalence of short sleep (41.2%) than married adults (30.8%). Psychosocial gradients were evident: individuals dissatisfied with life (53.8%) or reporting “never” receiving emotional support (51.5%) had nearly double the prevalence of short sleep compared with satisfied respondents (31.6%) or those who “always” received support (27.8%).

### 2.3. Multivariable Associations

#### 2.3.1. Sociodemographic and Psychosocial Predictors

In the fully adjusted complex samples logistic regression model (Table 1), several factors were independently associated with short sleep.

Education: Compared with college graduates, respondents with less than high school education had 15% higher odds of short sleep (AOR = 1.15, 95% CI: 1.08–1.23). High school graduates (AOR = 1.25, 95% CI: 1.20–1.29) and those with some college (AOR = 1.33, 95% CI: 1.28–1.38) also had significantly elevated odds.

Race/Ethnicity: Non-Hispanic Black adults had 56% higher odds (AOR = 1.56, 95% CI: 1.46–1.65), and American Indian/Alaska Native adults had 46% higher odds (AOR = 1.46, 95% CI: 1.29–1.65) of short sleep compared with non-Hispanic White adults. Hispanic adults (AOR = 1.08, 95% CI: 1.02–1.21) and Asian/Other adults (AOR = 1.14, 95% CI: 1.05–1.34) also showed increased risk.

Marital Status: Divorced/separated adults (AOR = 1.31, 95% CI: 1.23–1.40) and widowed adults (AOR = 1.21, 95% CI: 1.08–1.36) had greater odds of short sleep than married adults.

Sex: Males had slightly reduced odds compared with females (AOR = 0.94, 95% CI: 0.91–0.99).

Psychosocial Factors: Dissatisfaction with life was strongly associated with short sleep (AOR = 1.43, 95% CI: 1.33–1.54). Emotional support showed a dose–response gradient: compared with “always,” respondents reporting “rarely” receiving support had 68% higher odds (AOR = 1.68, 95% CI: 1.54–1.82). Social connectedness was protective: those with high connectedness had 32% lower odds of short sleep (AOR = 0.68, 95% CI: 0.62–0.75).

#### 2.3.2. Economic and Behavioral Predictors

When socioeconomic and behavioral factors were modeled (Table 2), additional associations were observed.

Income: A clear gradient was observed: compared with the highest income group, the lowest income group had 32% higher odds of short sleep (AOR = 1.32, 95% CI: 1.22–1.43), with intermediate groups showing progressively higher odds.

Smoking: Current smokers had nearly 70% higher odds (AOR = 1.69, 95% CI: 1.62–1.78), and former smokers also had elevated odds (AOR = 1.56, 95% CI: 1.45–1.68) compared with never smokers.

Binge Drinking: Binge drinking was not significantly associated with short sleep (AOR = 0.97, 95% CI: 0.93–1.00, *p* = 0.068).

Employment and Transportation: Not experiencing job loss was associated with slightly lower odds of short sleep (AOR = 0.93, 95% CI: 0.88–0.98), as was absence of transportation barriers (AOR = 0.80, 95% CI: 0.75–0.86).

Urban/Rural: Residence was not significantly associated with short sleep (AOR = 0.98, 95% CI: 0.94–1.03).

Age Patterns: Short sleep followed a nonlinear pattern across age groups. Adults aged 35–64 exhibited the highest adjusted odds of short sleep, with odds ratios > 2.5 compared to adults ≥ 80 years. Younger adults (aged 18–24 years) also had elevated risk compared with older adults, though estimates were smaller. When modeled continuously, increasing age was associated with slightly reduced odds of short sleep (AOR per year = 0.988, 95% CI: 0.987–0.989).

**Figure 1:**
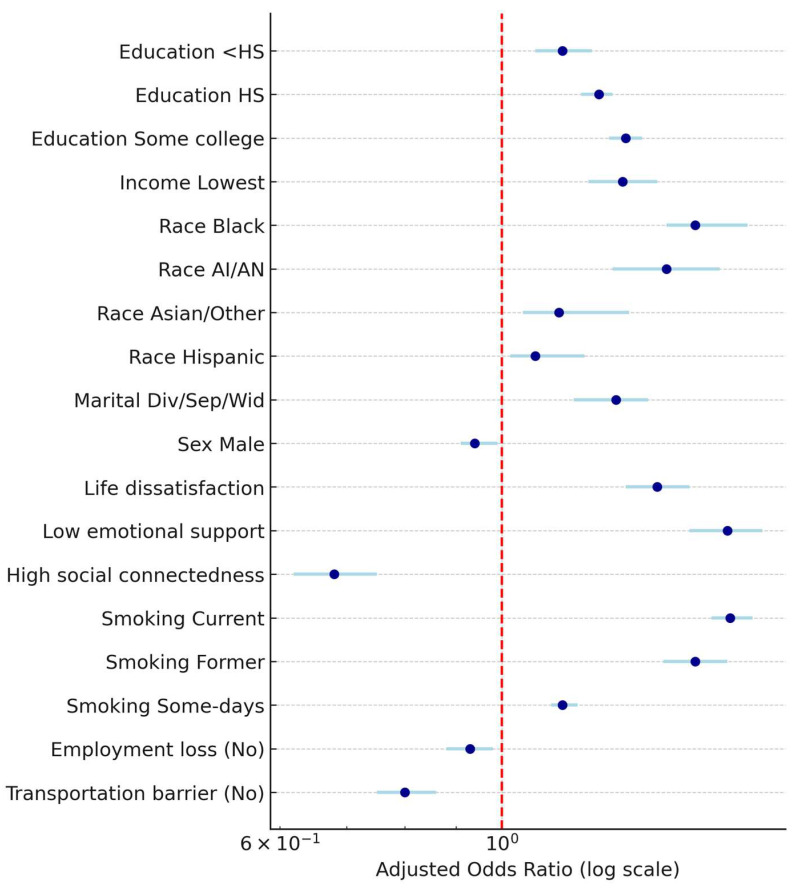
Adjusted odds ratios (AORs) and 95% confidence intervals (CI) for predictors of short sleep (≤6 h vs. ≥7 h).

Figure 1 shows the adjusted odds ratios (AORs) and 95% confidence intervals for predictors of short sleep (≤6 vs. ≥7 h) among U.S. adults, showing sociodemographic, psychosocial, behavioral, and structural determinants from the 2022 BRFSS.

## 3. Discussion

In this post-COVID-19 pandemic nationally representative analysis of BRFSS 2022 data, approximately one-third of U.S. adults reported short sleep duration (≤6 h), aligning closely with the CDC’s 2020 estimate of 33.2% [17]. Consistent with trends identified in both BRFSS and NHIS data, racial and ethnic disparities in short sleep persist. A NHIS analysis (2004–2018) found that non-Hispanic Black adults consistently experienced significantly higher short-sleep prevalence than White adults, with differences up to 10 percentage points, and these gaps widened over time across age and income groups [18]. In a UK Biobank analysis of nearly 500,000 adults aged 40–70, social deprivation and ethnicity were independently linked to worse sleep outcomes: 24.7% reported short sleep and 7.7% long sleep, both of which increased with deprivation, while White adults (72.4%) were more likely than Asian (65.4%), Mixed (63.8%), or Black (50.1%) adults to report recommended durations; a composite “Problematic Sleep Index” confirmed that poorer sleep clustered in non-White and socioeconomically deprived groups [19]. Similarly, our results point to robust associations between lower socioeconomic status indicators and increased odds of short sleep, consistent with economic deprivation’s known contributions to sleep disruption [20]. The midlife peak in short sleep prevalence, meaning those aged 35–64 had the highest adjusted odds relative to older adults. Adding an environmental dimension, the Southern Community Cohort Study of over 45,000 Black and White adults from low-income U.S. communities, found that higher neighborhood light-at-night exposure increased the odds of persistent long sleep (OR 1.23, 95% CI 1.02–1.48) and of shifting from long to short sleep (OR 1.35, 95% CI 1.06–1.72), while higher nighttime noise was associated with persistent short sleep (OR 1.19, 95% CI 1.07–1.31) and long-to-short sleep transitions (OR 1.31, 95% CI 1.05–1.64) [21].

Psychosocial stressors, particularly dissatisfaction with life, low emotional support, and social isolation, emerged as strong predictors of short sleep, independent of sociodemographic factors. This builds on the growing literature linking minority stress and psychosocial adversity to sleep health disparities, including among sexual and gender minority populations [22,23]. Social isolation (living alone, limited social interactions) and subjective loneliness have been shown to be associated with poor sleep quality, including increased sleep latency, fragmented sleep, reduced overall quality, and shorter sleep duration [24]. Mechanistically, Azizi et al. propose that social isolation acts as a chronic stressor, triggering HPA-axis dysregulation and heightened sympathetic activation, both contributors to impaired sleep regulation [24]. Another proposed mechanism is the feeling unsafe, lack of social presence may undermine perceived safety, leading to hypervigilance at night and sleep disruption [24]. Interestingly, a systematic review of seven polysomnography studies (n ≈ 7600 adults across the U.S., Switzerland, Brazil, and India) showed that lower socioeconomic status was associated with longer sleep latency, more wake after sleep onset, lower sleep efficiency, and more stage shifts. Individuals from low childhood SES backgrounds spent less time in slow-wave sleep, while financial strain predicted poorer PSG-assessed sleep continuity [25].

Beyond the United States, international research consistently supports the global relevance of social and structural determinants for sleep health. In Europe, a 23-country analysis demonstrated that lower satisfaction with living standards, financial worries, and adverse health conditions were strongly associated with higher reports of restless sleep [26]. In China, a nationwide study of over 500,000 adults showed that lower socioeconomic status, social isolation, and stressful life circumstances were strongly linked with higher odds of short sleep and insomnia symptoms, with about one in four participants reporting sleep ≤ 6 h per night [27]. A large national study of more than 95,000 community-dwelling older adults in New Zealand examined the links between loneliness, social isolation, and sleep problems [28]. At baseline, short sleep was more common than excessive sleep, and nearly one quarter of women and one fifth of men reported loneliness [28]. Interestingly, people living with others were more likely to report excessive sleep, suggesting different mechanisms at play [28]. In Brazil (São Paulo), three population-based surveys of adults (1987, 1995, 2007) showed striking increases in sleep complaints, with difficulty maintaining sleep rising from 15.8% in 1987 to 36.5% in 2007, and early morning awakening increasing from 10.6% to 26.7%. While social determinants were not specifically assessed, the authors suggested that social demands, economic pressures, and hectic lifestyles may have contributed to these trends, with greater awareness and knowledge about sleep also possibly influencing reporting [29].

Mechanistically, social stressors such as low socioeconomic status and chronic work overload disrupt sleep via alterations in the hypothalamic–pituitary–adrenal (HPA) axis, reducing slow wave sleep and REM sleep, and activating the sympathetic nervous system, which in turn impacts arousal and metabolism [30]. Rocha et al. showed that higher family income predicted longer sleep duration, while higher parental education was linked to shorter sleep duration but also less variability and shorter sleep latency. The authors noted a flatter diurnal cortisol slope, reflecting HPA axis dysregulation, suggesting that stress biology is one mechanism through which socioeconomic status influences sleep [31].

We found that urban/rural residence was not significantly associated with short sleep after adjustment, mirroring others who have shown similar results in other U.S. populations [32]. Smoking emerged as a strong behavioral risk factor for short sleep, consistent with well-established links between nicotine use and sleep quality and duration, with higher nicotine use and duration associated with shorter sleep [33].

Although there is an association between long commuting time and decreased sleep duration [34], one novel aspect in this study is the transportation barriers as a predictor of short sleep; existing work on transport mostly examines commute time or mode, not HRSN “transportation barriers.” Showing a protective association when not having transportation barriers and no job losses.

A major strength of this study is the use of BRFSS 2022 data, which provides a large, weighted sample, robust sociodemographic representation, and inclusion of psychosocial and health access variables. Moreover, complex-sample regression enhances the validity of national estimates.

While our findings confirm several previously reported disparities, replication in a large, updated national dataset is itself a valuable contribution. Replication strengthens confidence in established associations and is a cornerstone of cumulative science [35,36]. Furthermore, by analyzing BRFSS 2022, we provide timely estimates in the post-pandemic era, examine less frequently studied subgroups such as American Indian/Alaska Native adults, and incorporate novel structural factors (transportation barriers, job loss) alongside psychosocial determinants within the same model. Together, these features extend the literature on sleep health inequities and highlight the need for ongoing population studies on sleep health.

Limitations include reliance on self-reported sleep duration, which may introduce recall bias, and the cross-sectional design, which precludes causal inference. Although BRFSS includes a general measure of mental health through the “number of days in the last 30 days with poor mental health” item, it does not incorporate validated psychiatric assessments (PHQ-9 or GAD-7). As such, our ability to capture the full complexity of mental health influences on sleep is limited. Future studies should integrate more detailed assessments of mental health to provide deeper insights into sleep disparities. Future studies should augment with longitudinal designs, objective sleep measures (e.g., actigraphy), and more nuanced assessment of gender identity and minority stress factors.

## 4. Materials and Methods

### 4.1. Data Source and Study Population

We used publicly available data from the 2022 Behavioral Risk Factor Surveillance System (BRFSS), a nationally representative, state-based telephone survey of noninstitutionalized adults in the United States conducted by the CDC. The BRFSS collects health-related risk behaviors, chronic health conditions, and preventive service use among non-institutionalized adults aged ≥ 18 years across all 50 states, the District of Columbia, and participating U.S. territories. Detailed information about the survey design and weighting methodology is publicly available on the CDC website.

For this analysis, we restricted the sample to adults with complete data on sleep duration and covariates of interest, retaining the original variable names used by the CDC (“race/ethnicity”), and quantifying sleep in whole hours as surveyed. Respondents with missing, refused, or “don’t know” responses were excluded from regression analyses.

### 4.2. Measures

#### 4.2.1. Sleep Duration

Sleep duration was assessed using the BRFSS item: “On average, how many hours of sleep do you get in a 24-h period?” (SLEPTIM1). Consistent with AASM recommendations, responses were dichotomized as short sleep (<7 h) versus adequate sleep (≥6 h, reference category).

#### 4.2.2. Sociodemographic Characteristics

Demographic variables included age group (13 categories, collapsed for analysis into 18–24, 25–34, 35–44, 45–54, 55–64, 65–74, 75–79, ≥80), birth sex (female male), race/ethnicity (non-Hispanic White, non-Hispanic Black, American Indian/Alaska Native, Asian/Other, Hispanic), education (<high school, high school graduate, some college, college graduate), income (seven categories, highest income, marital status (married, divorced/separated, widowed, never married, member of unmarried couple, other), and urban/rural residence.

#### 4.2.3. Psychosocial Factors

Psychosocial measures included life satisfaction (satisfied/very satisfied vs. dissatisfied/very dissatisfied), emotional support (always, usually, sometimes, rarely, never), and social isolation (five levels, with most isolated as reference).

#### 4.2.4. Economic and Behavioral Factors

Food insecurity, loss of employment, and lack of transportation were coded as binary variables (yes vs. no). Health behaviors included current smoking status (never, current, former, some days) and binge drinking (yes vs. no).

### 4.3. Statistical Analysis

All analyses incorporated survey weights (_LLCPWT), strata (_STSTR), and primary sampling units (_PSU) to account for the complex BRFSS design. Descriptive analyses were conducted to estimate weighted prevalences of short sleep by sociodemographic and psychosocial characteristics. Complex Samples Logistic Regression (SPSS v31.0) was used to evaluate associations between predictors and short sleep, accounting for the BRFSS survey design (weights, strata, and primary sampling units). Adjusted odds ratios (AORs) with 95% confidence intervals (CIs) were reported. Statistical significance was set at α = 0.05 (two-tailed).

## 5. Conclusions

To our knowledge, this is among the first national analyses of the 2022 BRFSS to examine short sleep in relation to both psychosocial supports and health-related social needs (HRSN) within a single model. In particular, the protective associations we identified for the absence of transportation barriers and employment loss represent novel contributions to the sleep disparities literature, as prior national studies have rarely considered these structural factors in relation to sleep health. By integrating psychosocial connectedness and structural disadvantage, this study extends the existing literature on social determinants of sleep and underscores the importance of addressing both individual-level and structural barriers to improve sleep health equity.

Furthermore, our findings reinforce the need for multifaceted public health strategies that address both structural determinants, like educational access and income inequality, and psychosocial supports such as emotional and social connectedness. Clinicians should also consider screening for sleep issues in populations with elevated psychosocial or demographic risk profiles.

## Figures and Tables

**Table 1:** Sociodemographic and Psychosocial Predictors of Short Sleep.

Predictor	AOR	95% CI	*p*-Value
Education (<HS vs. College)	1.15	1.08–1.23	<0.001
Education (HS vs. College)	1.25	1.20–1.29	<0.001
Education (Some College vs. College)	1.33	1.28–1.38	<0.001
Income (Lowest vs. Highest)	1.32	1.22–1.43	<0.001
Race/Ethnicity (Black vs. White)	1.56–1.76	1.46–1.85	<0.001
Race/Ethnicity (AI/AN vs. White)	1.46–1.58	1.29–1.65	<0.001
Race/Ethnicity (Asian/Other vs. White)	1.14–1.25	1.05–1.34	<0.01
Race/Ethnicity (Hispanic vs. White)	1.08–1.16	1.02–1.21	0.005
Marital (Divorced/Separated vs. Married)	1.21–1.39	1.18–1.40	<0.001
Sex (Male vs. Female)	0.94–0.99	0.91–1.01	0.28–<0.001
Life satisfaction (Dissatisfied vs. Satisfied)	1.43	1.33–1.54	<0.001
Emotional support (Rarely vs. Always)	1.68	1.54–1.82	<0.001
Social connectedness (High vs. Isolated)	0.68	0.62–0.75	<0.001

Reference categories: College education, highest income, non-Hispanic White, married, female, satisfied with life, always emotional support, socially isolated.

**Table 2:** Health Behavior and Access Predictors of Short Sleep.

Predictor	AOR	95% CI	*p*-Value
Smoking (Current vs. Never)	1.69	1.62–1.78	<0.001
Smoking (Former vs. Never)	1.56	1.45–1.68	<0.001
Smoking (Some days vs. Never)	1.15	1.12–1.19	<0.001
Binge drinking (Yes vs. No)	0.97	0.93–1.00	0.068
Employment loss (No vs. Yes)	0.93	0.88–0.98	0.010
Transportation barrier (No vs. Yes)	0.80	0.75–0.86	<0.001
Urban vs. Rural	0.98	0.94–1.03	0.48

Reference categories: Never smoker, no binge drinking, employment loss, transportation barrier present, rural.

## Data Availability

Data is publicly available.

## Abbreviations

The following abbreviations are used in this manuscript:

AASM	American Academy of Sleep Medicine
AI/AN	American Indian/Alaska Native
AOR	Adjusted Odds Ratio
BMJ	British Medical Journal
BRFSS	Behavioral Risk Factor Surveillance System
CDC	Centers for Disease Control and Prevention
CI	Confidence Interval
HPA	Hypothalamic–Pituitary–Adrenal (axis)
HRSN	Health-Related Social Needs
HS	High School
JAMA	Journal of the American Medical Association
LLCPWT	Landline/Cell Phone Weight (BRFSS survey weight)
NHIS	National Health Interview Survey
OR	Odds Ratio
PSU	Primary Sampling Unit
REM	Rapid Eye Movement (sleep)
SPSS	Statistical Package for the Social Sciences
STSTR	Stratum (BRFSS survey stratum)
U.S.	United States
